# Thioredoxin: an antioxidant, a therapeutic target and a possible biomarker

**DOI:** 10.1038/s41390-024-03370-6

**Published:** 2024-06-28

**Authors:** Tara Sudhadevi, Anantha Harijith

**Affiliations:** Department of Pediatrics, Case Western Reserve University, Cleveland, OH USA

Study by Haga et al. on the utility of neonatal serum thioredoxin-1 (TRX-1) levels as a biomarker of bronchopulmonary dysplasia (BPD)/ retinopathy of prematurity (ROP) is the first of its kind. A biomarker is considered to enable the detection of a pathological condition or its severity and ideally, should have the capacity to be detected in the pre-disease state or during the diseased state. Premature infants who develop BPD are exposed to supplemental O_2_ and show marked evidence of oxidant stress as suggested by elevated glutathione disulfide (GSSG) concentrations.^1^ Upon preterm delivery, an infant breathing even atmospheric oxygen of 21% undergoes a sudden increase in lung O_2_ levels which perturbs the hypoxia-mediated signaling necessary for lung development in utero. Oxidative stress occurs in cells as a result of imbalance between the production and accumulation of reactive oxygen species (ROS).^2^ ROS are generated in cells routinely as by-products of oxygen metabolism. However, environmental stressors such as ionizing radiation, pollutants, heavy metals and exposure to hyperoxia greatly increase ROS production. Though ROS have physiological roles such as cell signaling, excessive accumulation results in oxidative stress and cell damage. Antioxidant systems such as superoxide dismutase, glutathione (GSH), glutaredoxin, heme oxygenase and thioredoxin (TRX) systems protect cells from the pathological effects of ROS.^3^ A deficiency in mounting efficient antioxidant responses to hyperoxia leads to increased susceptibility to oxidant injury. This is a problem seen in preterm infants who are more susceptible to the effects of oxidant injury due to developmental deficits in antioxidants.^4^ In this context, an understanding of the antioxidant systems is critical, and one such important system is thioredoxin.

**Fig. 1:**
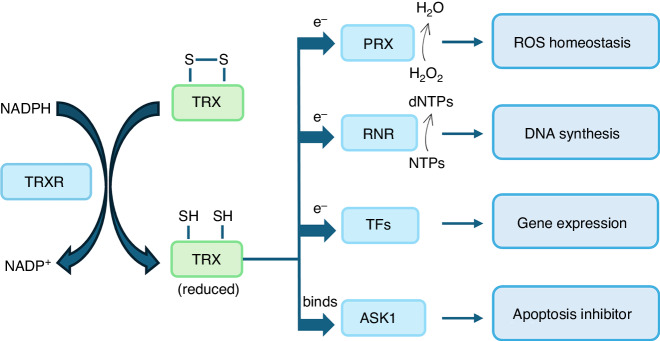
Working of the thioredoxin system. The NADPH produced by the pentose phosphate pathway donates electron to the thioredoxin (TRX) system keeping the TRX reductase (TRXR) in its reduced state, which in turn provides reducing equivalents to TRX. Reduced TRX donates electrons to several cellular proteins activating several cellular functions. Along with cellular peroxiredoxins (PRX) and ribonucleotide reductase (RNR), reduced TRX scavenges intracellular hydrogen peroxide (H_2_O_2_) and generates 2’‐deoxyribonucleotides (dNTPs) respectively. Reduced TRX also modulates the binding of transcription factors to DNA regulating gene expression. It also interacts with apoptosis signal-regulating kinase 1 (ASK1) and prevents apoptosis.

The thioredoxin (TRX) system comprises of NADPH, thioredoxin reductase (TRXR), and thioredoxin. This is an important antioxidant system against oxidative stress acting through its disulfide reductase activity regulating protein dithiol/disulfide balance. The TRX system donates electrons to the thiol-dependent peroxidases (peroxiredoxins) leading to the removal of reactive oxygen and nitrogen species.^5^ The enzyme TRX reductase maintains TRX in a reduced state (Fig. 1). TRX-1 is the most prominent protein in the TRX system and plays a critical role in various cellular functions such as maintenance of redox homeostasis, modulation of transcription factors, cell proliferation including DNA synthesis and control of cell death.^6,7^ As a protein playing a significant role in multiple cellular functions, modulation of TRX-1 expression and activity alters the metabolism from a physiological state to a pathological state leading to conditions such as cancer, and neurodegenerative and cardiovascular diseases.^8^ An interesting aspect of TRX-1 is that it is secreted into the extracellular space following oxidative stress and is detectable in significant concentrations in the body fluids such as plasma and bronchoalveolar lavage fluid, making it a good marker of oxidative stress. TRX-1 secreted into the extracellular space is mainly in the oxidized form. Secreted TRX-1 has chemotactic properties for monocytes, polymorphonuclear leukocytes, and T lymphocytes.^9^ TRX-1 is also induced by H_2_O_2_, ionizing irradiation, viral infection, and ischemia-reperfusion injury.

Intracellularly, the cytosolic concentrations of TRX family proteins are much lower compared to that of glutathione (GSH) but significant enough to provide additional reducing capacity to counter intracellular oxidative stress. Animal studies have shown that 95% hyperoxia substantially enhanced cellular ROS generation, increased apoptosis, induced TRX-1 and TRXR-1 expression while the TRXR-1 activity was reduced.^10^ The reduction in TRXR-1 activity may contribute to the increased redox injury to cells. In this context, it has been shown in animal models that recombinant human TRX-1 (rhTRX-1) provides cytoprotection by reducing ROS formation.^11^ An increased concentration of TRX-1 had a positive effect on the activity of other antioxidants such as manganese superoxide dismutase and GSH peroxidase.^12^ Animal studies overexpressing TRX such as the TRX transgenic mice (TRX-Tg) helped elucidate the role of TRX in suppressing inflammation. TRX-Tg neonatal mice exposed to hyperoxia showed an improved mean linear intercept length and increased number of secondary septa in lungs compared to the control mice. Inflammatory cytokines such as interleukin-6, monocyte chemoattractant protein (MCP)-1, and chemokine (C-X-C motif) ligand 2 mRNA expression levels were reduced in the lungs of hyperoxia-exposed TRX-Tg mice compared to controls.^13^ Importantly, TRX-Tg mice exhibited reduced macrophage infiltration in the lungs during recovery. A systemic overexpression of human TRX-1 specifically protected against hyperoxia-induced apoptosis in the cells of alveolar walls.^14^ In addition to protecting the neonatal mice, it has been shown that overexpression of TRX-1 also reduced hyperoxia-induced acute lung injury in adult mice accompanied by reduced protein leakage into the lungs, alveolar damage, focal alveolar hemorrhage, and hyaline membrane deposits in the lungs.^15^ Anti-inflammatory properties of TRX have been studied using lipopolysaccharide in animal models.^16^ Recombinant human TRX-1 reduced the rate of preterm delivery in mice induced by lipopolysaccharide and prevented an elevation of proinflammatory cytokines including TNF-α, interferon-γ, MCP-1, and IL-6 in the maternal serum. In addition to its protective effect in neonatal mouse models, TRX-1 also showed its protective effect in adult mouse models of COPD induced by cigarette smoke.^17^ TRX-1 ameliorated neutrophilic inflammation accompanied by a reduction in the release of granulocyte-macrophage colony-stimulating factor. Lung histology revealed prevention of progression of emphysema, indicating that TRX-1 could evolve as a novel therapeutic agent for exacerbation of COPD. Other models where TRX-1 showed its therapeutic impact was in a mouse model of irritant contact dermatitis induced by croton oil.^18^ Topically applied recombinant human TRX-1 suppressed the local inflammatory response evidenced by a reduction of cytokines and chemokines, such as TNF-α, IL-1β, IL-6, CXCL1, and MCP-1 in the dermal tissue.

TRX-1 has been used in various clinical conditions as biomarkers. The serum TRX-1 levels showed an increase during asthma exacerbation and a significant correlation between the serum TRX level and the serum eosinophil cationic protein. An inverse correlation was noted with forced pulmonary expiratory volume.^19^ Serum levels of TRX-1 were increased in patients with sepsis compared to healthy individuals, and it was noted that non-survivors showed even higher TRX-1 levels compared to survivors.^20^ TRX-1 levels have been found elevated in patients with acute lung injury both in the bronchoalveolar lavage and serum.^21^ Sections of lung tissue showed greater expression of thioredoxin in alveolar macrophages and type II epithelial cells. Increased TRX-1 levels have been shown as a potential tool for predicting favorable prognosis in patients with acute ischemic stroke.^22^ In patients with coronary artery disease, decreased serum TRX-1 levels were closely correlated with the extent and severity of coronary artery disease.^23^ In this context, an attempt was made by Haga et al. to identify TRX-1 as a biomarker for BPD. The study serves as a pilot study involving the measurement of serum TRX-1 levels at multiple time points. However, no relationship was noted between BPD and the TRX-1 levels. Serum TRX-1 levels were measured at various stages such as at birth, 10–20 days of life and at 36–40 weeks of post-menstrual age/gestation. Various studies have reported biomarkers associated with neonatal lung injury allowing the prediction of development of risk for BPD.^24^ Most of the studies that have reported biomarkers in BPD have been done in the initial few weeks of the preterm infant’s life.^25^ In this study, no difference was noted in the serum TRX-1 levels between controls and patients with BPD or ROP irrespective of the severity of illness. As noted by the authors, there is no established reference range for TRX-1 levels in the neonates. This is the first study measuring the serum TRX-1 levels in neonatal infants. The levels of TRX-1 were noted to be much higher in neonates compared to healthy adults. The median of the serum TRX-1 levels in the extreme preterm infants was much lower at 153 (74.5–242) compared to the control which was 267 (150–428) ng/mL. This suggested that TRX-1 levels are developmentally regulated as the levels decline to that of normal adult levels of 10–30 ng/mL by 36–40 weeks post-menstrual age. Small sample size of patients, a large variation in the TRX-1 levels and the administration of prenatal drugs might have affected the serum TRX-1 levels obliterating its potential use as a biomarker in this study. Though the pilot study failed to detect the use of TRX-1 as a biomarker of BPD or ROP, this was the first attempt to do so. Future studies with larger samples may help delineate the significance of TRX-1 measurement in BPD or ROP and improve our understanding of this important molecule from a development point of view.

There are key limitations in using total Trx1 as a biologically relevant biomarker, which was not addressed sufficiently and that needs consideration. The overall pool of Trx1 exists as reduced, oxidized, and hyperoxidized forms. Antibody-mediated approaches like the Trx1 ELISA used by Haga et al. in this study, measures only the total Trx1 pool that includes Trx1-reduced and Trx1-oxidized, but do not enable identification of Trx1 redox state. Tipple et al. study used a different approach where they found no difference in the total lung Trx1 protein levels between room air and hyperoxia-exposed newborn mice when measured by conventional western blot. However, a significant shift in theTrx1-reduced/Trx1-oxidized ratio towards Trx1-oxidized was observed when measured using redox western blotting technique.^26^ Methods that distinguish between Trx1-reduced/oxidized within the serum are challenging to perform which necessitates development of new approaches. Additionally, the accuracy of Trx1 ELISA is an issue, and is dependent upon many conditions, as outlined in a study by Lundberg et al.^27^ These conditions, including the antibodies used and the recombinant human Trx1 standards utilized, impacted accuracy of measurement and contributed to huge differences in Trx1 levels between studies, making between-study comparisons very difficult. This concept is especially important given the between-study comparisons in the Haga et al’s manuscript. Importantly, analyses of human plasma and cerebrospinal fluid in their study without preventing heterophilic antibody interferences were likely to cause false positive results and obscure the obtained data.^27^ Overall, the results of both studies suggest technical and methodological improvements in order to use Trx1 as a biological marker for BPD.
